# Alternative mRNA Splicing and Promising Therapies in Cancer

**DOI:** 10.3390/biom13030561

**Published:** 2023-03-20

**Authors:** James D. Fackenthal

**Affiliations:** Department of Biological Sciences, College of Science and Health, Benedictine University, Lisle, IL 60532, USA; jfackenthal@ben.edu

**Keywords:** alternative mRNA splicing, cancer, targeted therapies

## Abstract

Cancer is among the leading causes of mortality worldwide. While considerable attention has been given to genetic and epigenetic sources of cancer-specific cellular activities, the role of alternative mRNA splicing has only recently received attention as a major contributor to cancer initiation and progression. The distribution of alternate mRNA splicing variants in cancer cells is different from their non-cancer counterparts, and cancer cells are more sensitive than non-cancer cells to drugs that target components of the splicing regulatory network. While many of the alternatively spliced mRNAs in cancer cells may represent “noise” from splicing dysregulation, certain recurring splicing variants have been shown to contribute to tumor progression. Some pathogenic splicing disruption events result from mutations in cis-acting splicing regulatory sequences in disease-associated genes, while others may result from shifts in balance among naturally occurring alternate splicing variants among mRNAs that participate in cell cycle progression and the regulation of apoptosis. This review provides examples of cancer-related alternate splicing events resulting from each step of mRNA processing and the promising therapies that may be used to address them.

## 1. Introduction

The Human Genome Project reached impressive milestones in 2003, 2021, and 2022 toward the complete sequencing and ordering of three billion base pairs of sequence [1,2,3]. Analysis of the sequence has revealed a surprisingly small number of candidate protein-coding genes relative to the number of suspected proteins in the proteome. The current number of protein-coding genes currently stands below 20,000 [2]. One potential contributor to the large diversity of enzymatic functions despite the paucity of protein-coding genes is alternative splicing, whereby a single pre-mRNA transcribed from a protein-coding gene can be alternatively spliced into a number of different, albeit related, proteins.

It has been estimated that 95% of protein-coding genes in the human genome are represented by alternative splicing products [4,5,6]. While there are numerous examples of regulated alternative splicing events that represent the spectrum of a given gene’s functions, it is not clear how many of the total alternative splicing events detected transcriptome-wide are functional and how many represent “noise” from the error-prone splicing processes [7,8,9]. Moreover, it is not clear how often differences in alternative splicing products seen between cell types are due to differences in alternative splicing events or differences in the stabilities of alternatively spliced mRNAs.

In recent years, it has become clear that alternative splicing patterns can contribute significantly to cancer risk, initiation, progression, and therapy response. According to a recent study, there were 23.6 million new cases and 10 million cancer deaths in a single year worldwide, representing a greater than 20% increase in both categories over the previous nine years [10]. Challenges in addressing cancer burdens include the numerous genetic, epigenetic, and environmental factors contributing to cancer development. It is now clear that alterations in mRNA splicing patterns are among the contributors to cancer incidence and progression. In some cases, single base changes that disrupt the normal splicing pattern of a tumor suppressor gene may be pathogenic and result in increased cancer risk. In other cases, somatic mutations in genes required for regulating transcriptome-wide mRNA splicing patterns can be associated with cancer progression. In yet other cases, naturally occurring alternative splicing patterns of numerous genes can be coopted by malignant cells to promote their own progression in the absence of any discernable mutations. In this review, I will briefly discuss the origins of mRNA splicing that distinguish cancer cells from normal cells and the types of therapies that can be used to address pathogenic splicing patterns.

### 1.1. Splicing Regulation

Over 98% of introns in the human transcriptome are the so-called U2-type introns, following the “GU-AG” rules, referring to the first two and last two bases of the intron [11]. The minor U12 intron types will not be discussed further in this review. The pre-mRNA sequence requirements for binding the core enzymatic splicing proteins (the spliceosome) are surprisingly simple: the two 5′-most bases of the intron (the splice “donor”) are GU and the two 3′-most bases (the splice “acceptor”) are AG. Additionally, there is an adenine at the branch site required for lariat formation, followed by a polypyrimidine track near the splice acceptor. Other sequences surrounding these largely invariant sites, both in the introns and nearby exon sequence, are also critical to interacting with the spliceosome components, but they are somewhat more variable and may participate in determining the strength of a splice site.

**Figure 1:**
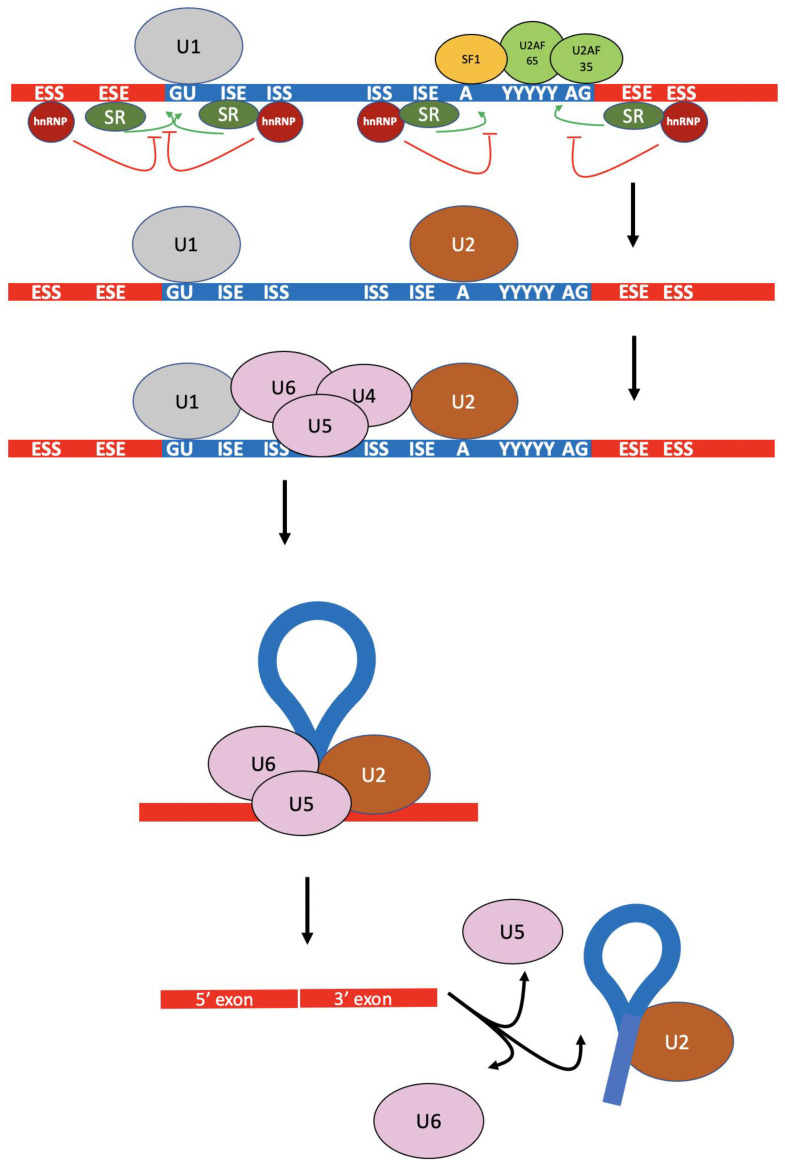
The multi-step mRNA splicing process shown for a single pair of exons. U2AF 35 and 65 proteins bind at the splice acceptor site and the polypyrimidine tract near the branchpoint adenine. The SF1 protein is bound at the site surrounding the branchpoint site, and the U1 snRNP is bound at the splice donor site. These activities can be promoted by SR proteins bound to splicing enhancers or inhibited by hnRNPs bound to splicing suppressors. SF1 and the auxiliary factors are supplanted by U2, followed by the binding of the U4/U5/U6 trimer. The intronic lariat is formed as a 5′ to 2′ phosphodiester bond between splice donor guanine and the branchpoint adenine as U1 and U4 leave the spliceosomal complex. The 5′ exon then joins the 3′ exon as the lariat is released.

Splicing is a multi-step process involving two transesterification reactions mediated by a series of complexes between pre-mRNA (the primary transcript), small nuclear RNAs (snRNAs) in the context of small nuclear ribonucleoprotein particles (snRNPs), serine/arginine-rich (SR) proteins, and other proteins. The snRNA components direct specificity of the snRNPs binding to the splice junctions of the pre-mRNA by base pairing (Figure 1). The first complex to form involves the U1 snRNP binding the 5′ splice site, while the U2 auxiliary factor (a heterodimer of U2AF65 and U2AF35) binds the polypyrimidine track adjacent to the branch point. The mammalian branchpoint binding protein (mBBP, or SF1) binds the sequence surrounding the branch site adenine. After this first “commitment” complex is established, the rest of the spliceosome is assembled in a specific series of events. The SF1 and U2AF proteins are replaced by the U2 snRNP, which binds to the branch point sequence. A trimer of U5, U4, and U6 snRNPs then joins the complex. Afterward, U1 leaves the complex, allowing U6 to bind the 5′ splice site. After the release of U4, a conformational change takes place that begins the first transesterification reaction, the joining of the 5′ guanine at the splice donor to the 2′ carbon on the branch point adenine site, forming a lariat structure. Further conformational changes take place that allow the second transesterification reaction to take place, using the 3′-most nucleotide of the “upstream” exon to join the first nucleotide of the “downstream” exon, releasing the lariat-shaped intron (reviewed in Wilkinson et al.) [12].

The invariant sequences bound by the core splicing proteins are not complex and can arise in multiple locations within the long introns of the pre-mRNAs typical of higher eukaryotes. Several other cis- and trans-acting factors participate in determining where and under what conditions the spliceosomes assemble. One factor is the order of emergence from the RNA Pol II. While splicing can occur on pre-mRNAs in vitro, in reality, splicing occurs at the same time as transcription. Indeed, the RNA polymerase II carboxy-terminal domain (CTD) interacts physically with a number of factors required for mRNA processing, including those required for 5′ cap formation, splicing, and even 3′ polyadenylation. This facilitates the assembly of spliceosomal components as they emerge from the RNA polymerase [13,14].

The core spliceosomal complexes are further directed to the correct positions on or near the splice junctions by serine/arginine-rich (SR) proteins bound to short variable sequences known as exonic splicing enhancers (ESEs), which define the positions of exons, or intronic splicing enhancers (ISEs), which define the position of introns. Conversely, core spliceosomal factors may be inhibited from binding at a given exon’s splice sites by heterogeneous nuclear ribonucleoparticles (hnRNPs) bound to short, variable sequences known as exonic splicing suppressors (ESSs) or intronic splicing suppressors (ISSs) [15,16]. These are the mediators of splice site identification and alternative splicing regulation.

**Figure 2:**
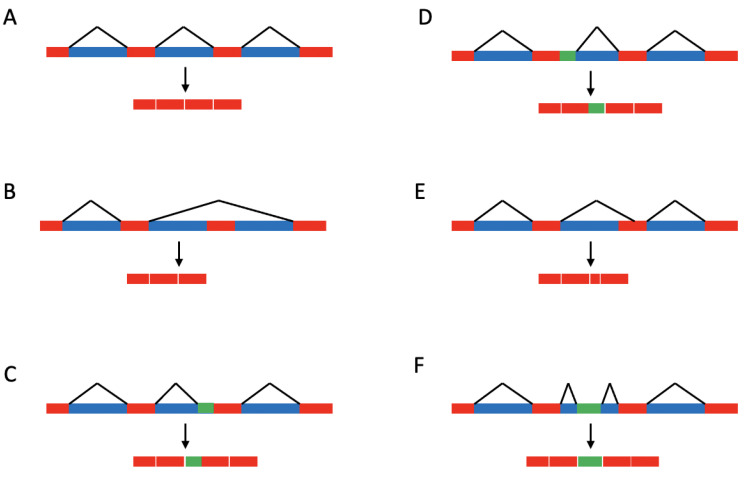
Commonly observed alternative splicing patterns of pre-mRNAs. (**A**). Normal splicing. The upper figure represents pr-mRNA with exons (red) and introns (blue). The lower figure represents mature mRNA consisting of joined exons following the splicing pattern indicated by the black lines. (**B**). Exon skipping. (**C**). An alternate splice acceptor site within an intron resulting in added sequence to the mature mRNA (green). (**D**). An alternate splice donor site within an intron. (**E**). An alternate splice acceptor within an exon resulting in a truncated exon in the mature mRNA. Note, alternate exonic splice donors also exist. (**F**). “Exonization” of a sequence within an intron resulting in an extra exon within the mature mRNA.

A single pre-mRNA may be alternatively spliced into a number of different isoforms (Figure 2). In many cases, the alternatively spliced pre-mRNA may undergo exon skipping, during which one or more exons are excluded from the mature mRNA. In other cases, exons may include alternate splice donor or acceptor sites, changing the number of codons they contain. In still other cases, small portions of intron sequence may be “exonized” and become included in the mature mRNA. Many such events occur in a regulated manner in response to environmental or developmental cues (reviewed in Diederichs et al., 2016 [17]).

When regulated, alternative splicing may serve several purposes. There are numerous examples of alternative splicing products translated into multiple proteins in the spectrum of single-gene products. For example, the Bcl-x protein, which participates in regulating apoptosis, can be translated from one of two alternately spliced mRNAs. The long isoform (Bcl-xL) is anti-apoptotic, and the short isoform (Bcl-xS) is pro-apoptotic. The splicing machinery that regulates the switch from one isoform to the other is responsive to DNA damage response pathways, thus connecting the survival of the cell to the state of DNA repair [18]. Naturally occurring alternative splicing events can also contribute to therapy resistance in some cancers. For example, melanomas associated with the oncogenic *BRAF* V600E mutation may become resistant to the V600E-specific drug vemurafenib by taking advantage of an alternative splicing event that removes the V600E-containing domain, which otherwise would have promoted aberrant signaling through the MAPK pathway [19]. It has recently been demonstrated that inhibition of the transcription factor FOXP2 may increase sensitivity to vemurafenib in *BRAF* V600E melanomas, suggesting a potential future therapy strategy [20]. Likewise, HI-511, which targets both AURKB and *BRAF* V600E, has shown promise as a therapy against drug-resistant melanomas in the future [21]. However, no strategies for directly targeting alternative splicing patterns of the *BRAF* V600E mRNA have been devised. Additionally, alternative splicing also affects the localization or stability of an RNA, regardless of whether it is translated. For example, incompletely spliced or incorrectly spliced mRNA may be retained in the nucleus or only inefficiently transported to the cytoplasm [22].

Processes resulting in correct or incorrect splicing can affect the array of proteins associated with mature and maturing mRNAs, including cap-binding proteins, poly-A binding proteins, exon junction complexes (EJCs), and assorted hnRNPs. These, in turn, may affect the rates and conditions under which RNA molecules are degraded by either nuclear or cytoplasmic exosomes or other RNA degradation pathways. For example, mRNAs with stop codons in any but the last exon (premature termination codons) will retain EJCs otherwise stripped from mRNAs by translation. Ribosomes dissociate from mRNA upon encountering termination codons, and the Upf proteins associated with the requisite termination factors trigger a degradation process if downstream EJCs remain, a process known as nonsense-mediated mRNA decay (NMD) [23]. Any alternative splicing event that alters the translational reading frame of the mRNA naturally has a high likelihood of introducing a premature stop codon and could thus affect the stability as well as the translatability of the message.

Trans-acting factors that affect alternative splicing patterns recognize post-transcriptionally modified RNA bases as well as primary sequence information. There are over 60 post-transcriptional modifications known to act on RNA nucleotides in eukaryotes, some of which can affect splicing [24,25]. Diseases including cancer have been found to be associated with mutations or the misregulation of factors that act as RNA “writers” (proteins that chemically modify RNA bases in the context of specific sequences or higher-order structures), “erasers” (enzymes that remove these modifications), and “readers” (proteins that bind specifically to modified or unmodified RNA base sequences or structures). As with DNA, many of the chemical modifications appearing on RNA are methylations of various positions on adenines or cytosines [24]. Such modifications could affect alternative splicing events, relative stabilities of potentially translatable RNAs, translation regulation, and RNA localization, all potentially affecting the physiological impact of alternatively spliced mRNAs. One RNA modification, N6-methyladenosine (m6A), has received considerable attention. That modified nucleotide in the context of pre-mRNA can be bound by the splicing factor YTHDC1, promoting exon inclusion [24,26]. The field of epitranscriptomics is fairly new and will doubtless shed light on the role of post-transcriptional mRNA modifications in tumor initiation and progression.

Alternative splicing, whether stochastic or regulated, is thus influenced by multiple events, providing multiple avenues for contributing to the initiation or progression of cancer. Indeed, cancer cells have been shown to have distinctive profiles of alternatively spliced mRNAs and expression levels of splicing regulators [27,28,29]. It has been demonstrated that alternate splicing events from splicing dysregulation can contribute to cancer development [30,31] and therapy resistance [32], and an understanding of alternative splicing mechanisms in cancer cells has led to promising avenues of research into therapies directed against alternative splicing processes and products. Several excellent reviews have described alternative splicing events as they relate to specific physiological systems or therapeutic strategies for targeting alternative splicing events in cancer [4,33,34,35,36,37]. In this review, I aim to focus on the origins of alternative splicing events themselves that may be common to multiple physiological systems.

### 1.2. Single-Base Changes Affecting Splicing of Disease-Associated Pre-mRNAs

Alternative splicing driven by mutations in cis-acting splice regulators plays a significant role in cancer development. It has been estimated that 14% of pathogenic point mutations affect splice sites in individual disease-associated genes and that as many as 50–60% of all mutations affect splicing in some way [38,39,40,41,42]. Numerous mutations in cancer-associated genes in particular have been associated with single base substitutions in consensus splice sites, ESEs, ESSs, ISEs, or ISSs [43]. For example, mutations that affect splicing have been found in all genes routinely screened for cancer predisposition, including *APC*, *ATM*, *BRCA1*, *BRCA2*, *BRIP1*, *CHEK2*, *CDH1*, *MLH1*, *MSH2*, *MSH6*, *PMS2*, *MUTYH*, *NF1*, *PTEN*, *PALB2*, *RAD51C*, *RAD51D*, and *TP53* [44] (and references therein).

**Figure 3:**
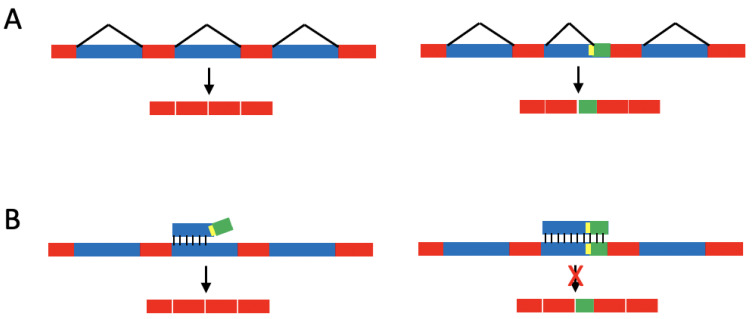
Allele specific oligonucleotides (ASOs) can form either DNA:DNA or DNA:RNA duplexes. In cases where one allele has an intronic or exonic base change (yellow) that generates an alternate splice site (**A**), the ASO can bind by base pairing to block splicing at that site (**B**). Mismatched bases will not allow the ASO to bind to the wild type sequence. Alternately, the ASO could bind to intronic sequences containing cis-acting regulatory sites in wild type sequences to alter the relative abundances of naturally-occurring alternative splicing variants.

Several approaches are being explored to address malignancies associated with single-base splicing mutations in disease-associated genes. One is to employ targeted antisense oligonucleotides (ASOs), which involves designing short single-stranded DNA or RNA oligonucleotides (12–28 bases) that are reverse complements to specific RNA sequences and base pair to form DNA/RNA or RNA/RNA duplexes. DNA/RNA heteroduplexes can promote nuclear degradation of the RNA by RNase H, prevent the loading of the RNA onto ribosomes in the cytoplasm, or interfere with splicing mechanisms to promote the production of specific alternate splicing products [45,46]. RNA/RNA heteroduplexes may be used to interfere with splicing patterns specifically (see Figure 3).

This approach for shifting mRNA splicing patterns has been developed to treat diseases such as Duchenne muscular dystrophy [43], and similar approaches are being developed to address some cancers. In one example, it has been demonstrated that the expression of the oncogenic KRAS(Q61K) allele, which occurs in multiple cancers, depends on the presence of the nearby G60G silent substitution to eliminate a cryptic splice donor site. A mutation-specific oligonucleotide directed against the ESE-rich region around codon 61 in the KRAS gene has been developed to alter the KRAS splicing patterns and prevent the accumulation of the KRAS(Q61K) protein [47].

Another ASO in development is aimed at altering the metabolic state of liver cancer cells by altering the balance between pyruvate kinase isoforms encoded by splice variants of the M2 pyruvate kinase gene (*PKM*), which is highly expressed in most cancers (Reviewed by Peng et al. [34]). In this case, the ASO-targeted splice variant of the *PKM (PKM2)* is a naturally occurring splice variant that does not result from a spliceogenic mutation. An anti-PKM2 ASO has been developed to shift the isoform balance away from the cancer-associated PKM2 isoform toward the pro-apoptotic isoform in glioblastoma cells [36].

Likewise, a naturally occurring (i.e., mutation-independent) splice variant that results in a truncated variant of the androgen receptor (*AR-V7*) is associated with castration-resistant prostate cancer. An antisense oligonucleotide designed to target an ISE of the *AR* pre-mRNA can shift the isoform balance toward full-length androgen receptor proteins in prostate cancer cells and can re-sensitize cells to androgen depletion [48]. ASOs may eventually become valuable components of therapeutic strategies as technologies progress.

In cases in which recessive mutations in consensus splice site sequences contribute to a disease, more targeted approaches may be employed to directly “correct” the resulting splicing defect. For example, mutated splice sites may be corrected by engineering corrective snRNPs with altered sequences that base-pair with the mutated splice sites. Exon-specific U1 snRNAs (ExSpeU1s) have been designed to restore correct splicing patterns to mutated mRNAs associated with spinal muscular dystrophy, propionic acidemia, and other disorders. While preclinical studies of these complexes have shown promise in other disease systems [35], they remain a largely unexplored strategy for cancer treatment.

### 1.3. Mutated and Dysregulated Splicing Factors

With respect to therapeutic strategies, transcriptome-wide dysregulation of splicing may be compared with the genomic instability seen in tumors: a limited amount may not be lethal to the transformed cell, and most events may be considered “noise,” but some events may provide a selective advantage to malignant cells. Therapies that target transcriptome-wide splicing-related events may, therefore, target mutated or overexpressed trans-acting splicing regulators, or splicing in general, with the aim of pushing transcriptome-wide mRNA dysregulation over the threshold to physiological unsustainability.

Mutations and amplifications of genes encoding trans-acting splicing factors are found in many cancers and could provide therapy targets by several means. In some cases, cancer progression may depend on aberrant splicing products of disease-associated genes generated by mutated splicing factors, which can be targeted directly. In other cases in which genes for trans-acting splicing regulators are mutated, malignant cells may depend on normal splicing patterns but are extra sensitive to therapies directed against the remaining wild-type allele [43,49]. In yet other cases, tumors are able to take advantage of naturally occurring altered splicing patterns without requiring mutations or the amplification of genes encoding splicing regulators.

Alternative splicing patterns that can promote cell cycle progression [50,51] or inhibit apoptosis [51,52] can be achieved by changing activity levels of non-mutated splicing regulators [49,53]. For example, the oncoprotein MYC promotes overexpression of the splicing regulators hnRNP A1 and hnRNP A2 [54] and PRMT5, an arginine methyltransferase that methylates and regulates some components of the U2 snRNP [55]. This suggests that MYC-dependent cancers may be susceptible to inhibitors of PRMT5 and other splicing regulators that may be overexpressed in MYC-driven cancer cells [56,57]. Pharmacological inhibition of PRMT5 has been shown to inhibit cancer-specific splicing patterns and inhibit cancer cell growth [55,57,58].

In some colorectal cancers, there is an overexpression of a pre-mRNA processing factor that is a component of the U5 snRNP (*PRPF6*), which is required for interaction with U4 and U6. This overexpression co-occurs with the alternative splicing of several mRNAs, including the mRNA for the signal transduction participant ZAK kinase. The high levels of PRPF6 correlate with increased levels of the long-form of the ZAK kinase mRNA (ZAK-LF), which promotes cell cycle progression and is often upregulated in cancers [59].

By contrast, other trans-acting splicing regulators can contribute to cancer progression when under-expressed or mutated. For example, the U5 component *PRPF8*, required for a catalytic step in pre-mRNA splicing, has functionally reduced protein levels in many myelodysplastic syndromes because of mutations or deletions. This results in transcriptome-wide mis-splicing involving increased use of sub-optimal splicing sites [60]. Likewise, the core spliceosomal associated genes *U2AF1* (which encodes U2AF 35) and *U2AF2* (which encodes U2AF 65) are mutated in some blood cancers, resulting in mis-splicing of numerous pre-mRNAs. Clinical applications of these observations remain opportunities for exploration.

Many myelodysplastic syndromes are associated with hotspot mutations in the gene encoding *SF3B1*, a protein associated with the U2 snRNP that participates in recognizing and selecting the intron branch site, resulting in widespread mis-splicing of pre-mRNAs [61,62]. For example, *SF3B1* mutations can be associated with the alternative splicing of the *TAL1* transcription factor, which results in reduced erythroid differentiation in vitro [61]. *SF3B1* mutations have also been detected in chronic lymphocytic leukemia (CLL), in which they are associated with potential alternative splicing events associated with anti-apoptotic functions [63,64].

Mutations in non-core spliceosomal components are also associated with altered splicing patterns distinctive to cancer cells. For example, the gene for SR protein SRSF2 (also called SC35), required for both alternative and constitutive splicing, has been found to be mutated in some hematological malignancies and associated with the mis-splicing of many pre-mRNAs [61,65,66].

While many malignancies are associated with mutated or amplified genes encoding splicing regulators, others are characterized by changes in relative frequencies of naturally occurring splice variants that may be shifted by environmental and metabolic conditions. For example, some therapeutic DNA-damaging agents have been shown to affect alternative splicing patterns of genes involved in promoting apoptosis, thus potentially contributing to their therapeutic effects. In one case, the DNA-damaging agent oxaliplatin was shown to change the binding activity of the SR protein SRSF10, the hnRNPs A1/A2, and the RNA-binding protein Sam68. These splicing regulators bind the Bcl-x pre-mRNA and have the therapeutically beneficial effect of increasing levels of the pro-apoptotic alternate splicing isoform Bcl-xS at the expense of the anti-apoptotic isoform Bcl-xL [67,68].

The DNA damage repair genes BCL2L1, BRCA1, CHEK2, and TNFRSF10B, are also subject to alternative splicing in response to oxaliplatin by the altered activities of SRSF10, the hnRNPs A1/A2, and Sam68 [30,67,68], potentially affecting their intended benefits. For example, there exists a splice variant of the BRCA1 DNA-damage repair gene (BRCA1-∆11q) that, at increased levels, can contribute to resistance to the DNA-damaging agent cisplatin as well as PARP inhibitors [69].

As altered activities of trans-acting splicing regulators are associated with the altered splicing patterns of mRNAs involved with apoptosis and DNA repair in cancer cells, small molecules that modulate the activity of splicing regulators have been developed as potential cancer therapies. Some of the earliest small molecule splicing inhibitors, such as pladienolide B (PB), its analog E1707, the E1707 analog H3B-8800, spliceostatin A, and sudemycins, act directly on the U2-associated protein SF3B1, [37,57,70,71,72]. Likewise, isoginkgetin is used to prevent the U4/U5/U6 tri-snRNP from joining the spliceosome, which blocks the progression of the splicing pathway [37,73,74] (Table 1).

As potential therapies, general splicing inhibitors are blunt instruments. They could potentially push cells with already compromised splicing fidelity over a threshold of splicing failure, resulting in tumor cell lethality, while inflicting only tolerable first “hit” damage to healthy cells. Indeed, cancer cells have been shown to be more sensitive to small molecule inhibitors of global splicing factors than non-cancer cells [85,86].

Several promising small molecule inhibitors of certain splicing events are being explored for potential therapeutic benefit. For example, the non-POU domain-containing octamer-binding protein (NONO) participates in splicing regulation as well as many other functions. It has been shown to be overexpressed in many cases of glioblastoma multiforme (GBM) and is required for the correct splicing of the glutathione peroxidase 1 (*GPX1*) gene, which is required for full levels of tumor growth and invasion. Tissue culture cells that lack NONO expression exhibit intron retention of the GPX1 pre-mRNA. The small molecule drug auranofin, normally used to treat rheumatoid arthritis, has been shown to target NONO, and may, therefore, be a potential candidate for treating GBM [80,81].

It has also been shown that indisulam and other reagents can target RBM39, a major component of a protein complex that is upregulated in AML and required for correct splicing of several pre-mRNAs required for AML cell survival with relatively little effect on normal cells [87]. Likewise, inhibitors of the PRMT protein arginine methyltransferases, such as GSK591 and MS023, have been shown to disrupt normal splicing in leukemias by inhibiting the regulation of several splicing factors. Leukemias carrying mutations in any of a number of genes for these splicing factors seem to be especially sensitive to PRMT inhibitors, suggesting a mechanism for cell type specificity and a means by which patients can be screened for potential therapeutic benefits [3]. Additionally, hematologic malignancies with mutations in SF3B1 or other members of the SF3b complex are especially sensitive to the SF3b complex-interacting compound H3B-8800 and cause the inhibition of both normal and aberrant splicing events promoted by SF3b complex mutations [57,88].

Finally, the complex lipids known as ceramides have also been shown to have unexpected roles in regulating splicing events relevant to apoptosis. Both the Bcl-x and Caspase 9 mRNAs can be alternatively spliced to generate either pro-apoptotic or anti-apoptotic variants. Ceramides have been shown to promote the dephosphorylation of SR proteins, resulting in an increase in the pro-apoptotic splice variants Bcl-xS and Caspase 9a and a decrease in the anti-apoptotic splice variants Bcl-xL and Caspase 9b in lung adenocarcinoma cells [89,90].

## 2. Summary

Considerable attention has been given to genomic dysregulation in cancer cells by mutations and epigenetic modifications. It is now clear that malignant cells can also take advantage of alternate mRNA splicing patterns, potentially providing further diagnostic molecular markers and targets for therapy. Many cancers associated with altered splicing patterns may begin with single base changes in disease-associated genes that fall within cis-acting splicing regulators. These can include base changes traditionally considered neutral with respect to the amino acid encoded. Transcriptome-wide dysregulation can occur when mutations occur in the genes encoding trans-acting splicing regulators. In some cases, this results in generating an imbalance of splice variants of several genes that can promote cell cycle progression or inhibit apoptosis. In other cases, cancer cells with a single inactivated splicing regulator gene may depend on normal splicing patterns to survive, but they will be extra sensitive to therapies directed against the remaining wild-type copy. In yet other cases, the balance of naturally occurring alternate splicing variants of mRNAs associated with cell cycle progression or inhibition of apoptosis can be shifted by alternate regulation of splicing regulators in the absence of mutations. Each source of cancer-associated altered splicing pattern presents opportunities for testing aimed at individualized therapy. Spliceogenic mutations resulting in the inactivation of single genes or transcriptome-wide dysregulation of splicing are currently or potentially addressed with allele-specific ASOs or ExSpeU1s. In cases in which cancer cells depend on wild-type splicing or a shift in the balance of naturally occurring alternate splicing variants, small molecules that target trans-acting splicing regulators may push malignant cells into a state of metabolic unsustainability and cell death. The ability to add diagnostics and therapies aimed at mRNA splicing to the arsenal currently targeting chromatin and protein activity holds great promise for cancer therapy in the future.

## Figures and Tables

**Table 1:** Small molecule inhibitors of trans-acting splicing factors.

Drug	Target	Status	References
Pladienolide	SF3B1	Preclinical studies in gastric cancer	[75]
E1707	SF3B1	Tested in Phase I clinical trials on myelodysplastic syndrome and several solid tumors. Not recommended for further testing.	[76,77]
H3B-880	SF3B1	Tested in Phase I on myeloid neoplasms. Not recommended for further testing.	[78]
Spliceostatin A	SF3B1	Rhabdomyosarcoma cell line	[71]
Sudamycin	SF3B1	Preclinical studies	[71]
Isoginkgetin	U4/U5/U6	Preclinical studies	[73,74]
Auranofin	Thioredoxin reductase, the ubiquitin-proteasome system, NONO	Approved for rheumatoid arthritis, in phase I/II clinical trials for several cancers.	[79,80,81]
Indisulam	RBM39	Completed phase II combination study in AML and myelodysplastic syndrome.	[82]
GSK591	PRMT	Preclinical studies in glioblastoma and leukemia	[3,83]
MS023	PRMT	Preclinical studies	[3,84]

## Data Availability

Data from previously published works described in this review are available as described in the references.
